# Hospitals and the Aristocracy of the Poor

**Published:** 1903-01-24

**Authors:** 


### HOSPITALS AND THE ARISTOCRACY OF THE POOR.

AT the meeting at the New Pay Hospital, Fitzroy House, W., on the 9th inst., an interesting point was discussed which nearly affects the present relations of all classes to the general hospitals in London. If the change of system indicated can be brought about we shall hear little or nothing of hospital abuse after it becomes universal.

### THE SELF-RESPECTING WORKMAN.

The Bishop of Southwark said as a "Slum Bishop," who for over a quarter of a century had worked among the poor of all classes, including the upper poor (for there were as many classes among those called "poor" as in other strata of society), he would be very glad if he could see this principle of self-help carried out among them. The King and those associated with him in his Hospital Fund had done wonders in helping the hospitals, but he did not feel it to be right that so many people should accept charity to the extent they did from the great hospitals. Men who were earning good wages were not only able but proud to take their part in the expenditure, and if it were put before them he believed they would rise to that higher idea. By a system of part payment, large sums of money for fresh ventures in hospital work among those who really needed charity would be set free. He would be glad if the principle were extended a long way beyond this house. His last word should be upon the sacred part of the meeting. He looked upon the dedication as a demonstration of what many men had come to think seriously about, viz, the real unity between the medical art and religion. There was no jealousy between the two. He had often stood with the medical man at the last supreme struggle of men's lives, and he was perfectly certain of this, that each had found that there was a place for the other. If he had ever had any success with one whom he might call a "spiritual patient," he realised that it had been made possible by the ministrations of the doctor, who enabled him to do his part. And he had known medical men who had said that the work of healing the body had often been helped by the healing of the soul. He hoped that, in the case of Fitzroy House, the healing of the body might be helped by those forces which give hope, which, after all, were forces that came from religion.

### HOSPITAL PAY WARDS.

Sir Henry Burdett said the Bishop of Southwark had spoken of the aristocratic poor. He (the speaker) had known for years that there was a great demand among the upper classes of poor—*e.g.* artisans and clerks—for the privilege of paying for hospital treatment, and he had always felt that it was a hardship that they should not be permitted to do so. The spirit of dependence fostered by the present hospital system was a source of danger, inasmuch as it was likely to affect the character of the nation. To treat all hospital patients alike, because we were either too lazy or too incapable to discriminate, was a bad system; each patient should have his rights, while at the same time the medical profession should be protected. The great difficulty had been the permanent debt of £100,000 on the London hospitals, which had never been overcome. It had gone on from year to year. Now, however, the King's Fund had completely altered this state of affairs; the accumulated debt had been removed, and the opening of this pay hospital marked a departure in the history of hospital administration, by proving that the principle was financially sound, it put every hospital in a position to consider the question of paying wards. He had no doubt that if the public—and he interpreted the beginnings of the desire for it—had the matter brought home to them, the accommodation would speedily be forthcoming in the form of paying wards.

After alluding to a provident scheme which he has in hand for enabling everyone to procure six weeks' hospital treatment in pay wards by paying an annual premium, Sir Henry said that pay wards meant death to hospital abuse. He had seen the system at work on the Continent and in the United States, where by simply requiring each patient to prove that he was worthy of free treatment or to pay for it, all hospital abuses might disappear.

